# Heterosis and its potential influence on pulmonary arterial pressure in beef cattle^1,2^

**DOI:** 10.1093/tas/txaa117

**Published:** 2020-12-22

**Authors:** Roderick A González-Murray, Miguel A Sánchez-Castro, Milton G Thomas, Scott E Speidel, R Mark Enns

**Affiliations:** Department of Animal Sciences, Colorado State University, Fort Collins, CO

## INTRODUCTION

Hypoxic environmental conditions typical of high elevations (>1,524 m) produce a pathologic condition in cattle termed high-altitude disease (HAD). Cattle experiencing HAD manifest pulmonary vascular remodeling and pulmonary hypertension, which if not ameliorated by relocating to lower elevation, could lead to cardiopulmonary insufficiencies, right heart failure, and death (Holt and Callan, 2007; Jennings et al., 2019). Although the prevalence of HAD in cattle raised in high elevations has been reported to be relatively small (5%), the negative economic impact related to death losses within the U.S. beef industry has been estimated to be as high of $60 million, considering that >1.5 million Western U.S. cattle are raised at high elevations (Williams et al., 2012).

The primary diagnostic tool to determine an animal’s susceptibility to HAD is the measurement of pulmonary arterial pressure (PAP). Factors contributing to variations in PAP include breed, gender, age, body condition, concurrent illness, environmental conditions, elevation, and genetics (Holt and Callan, 2007). From a genetic selection perspective, PAP is considered as an indicator trait with a moderate heritability, which suggests that possibilities to genetically reduce the incidence of HAD by using it as a selection criterion at high elevations are feasible (Shirley et al., 2008; Crawford et al., 2016; Pauling et al., 2018).

Although genetic improvement by direct selection on PAP is feasible due to the existence of adequate genetic variation for this trait, strategies such as heterosis may represent another avenue to reduce the incidence of HAD in beef cattle since its benefits tend to increase under stressful environmental conditions (Keller and Brinks, 1978; MacNeil et al., 1989). Previous reports using multibreed data suggested that breed represents a significant source of variation in yearling PAP measurements (Crawford et al., 2017). In this sense, composite animals could potentially be less susceptible to HAD; however, only one report has been made regarding the effects of heterosis on PAP measures in a multibreed beef cattle population (Culbertson et al., 2016). Therefore, objectives of this study were to determine heterosis and breed percentage effects on PAP measurements.

## MATERIALS AND METHODS

The data used in the present study were obtained from an existing database; therefore, the study was not subjected to animal care and use committee approval.

### Data Collection and Editing

Data containing 1,083 individuals with varying percentages of Simmental, Angus, Hereford and Red Angus breeds were used for the study. Information relative to sex, herd location, and its associated elevation, as well as PAP-related information (PAP observation, PAP measurement date, and PAP technician) was available for all animals. Parental information (e.g., breed percentage) of individuals with PAP observations was retrieved from a multibreed population database. Breed percentages of parents were utilized to estimate the degree of outcross of each individual with a PAP observation using the following formula (Bourdon, 2000):

$$\begin{array}{l} Degree\;of\;outcross=\left[1-\underset{i=1}{\overset{n}{\sum }}\;P_{s_{i}}P_{d_{i}}\right]*100 \end{array}$$

where $P_{s_{i}}\;$ represented the proportion of the ***i***th breed in sires and $P_{d_{i}}$ corresponded to the proportion of the ***i***th breed in dams.

### Statistical Analysis

A general linear model (GLM) was used to estimate the effect of heterosis on PAP. Contemporary group (defined as a combination between herd, PAP date, PAP technician, and sex) was included as a categorical fixed effect, whereas the age at PAP measurement and the degree of outcross were included as linear covariates. Similarly, breed effects were estimated by including breed percentages of Simmental, Angus, Hereford, Red Angus, and “Other breeds” as linear covariates in the model. All analyses were performed using the statistical software package ASREML 3.0 (Gilmour et al., 2009).

## RESULTS AND DISCUSSION

The general average for PAP measurements in the entire data set was 41.29 ± 0.22, with a minimum of 26 and a maximum of 108 mmHg. However, summary statistics for PAP and age at PAP measurement according breed group are presented in Tables 1 and 2, respectively. Considerable differences in the number of PAP records per breed group were observed as the Simmental group represented approximately 8.1% of the data, while the composite groups Simmental × Angus, Angus × Hereford, Angus × Red Angus, and Angus × Other breeds comprised 18.7%, 10.6%, 61.9%, and 0.7%, respectively. This data structure is a clear reflection of one of the challenges associated with the use of field data for multibreed analyses as acknowledged in previous reports (Legarra et al., 2007; Golden et al., 2009).

**Table 1.** Summary statistics for pulmonary arterial pressure (mmHg) according to breed group

Breed group	*N*	Average	SD	Min	Max
Simmental	85	39.75	4.09	34	63
Simmental × Angus	203	40.04	3.77	32	52
Angus × Hereford	115	38.73	5.10	28	54
Angus × Red Angus	672	42.32	8.44	26	108
Angus × Others	8	39.25	5.14	34	51

**Table 2.** Summary statistics for age (d) at pulmonary arterial measurement according to breed group

Breed group	*N*	Average	SD	Min	Max
Simmental	85	371.13	97.78	168	543
Simmental × Angus	202	259.97	14.81	215	314
Angus × Hereford	115	308.70	41.70	231	419
Angus × Red Angus	668	334.82	49.33	166	444
Angus × Others	8	335.88	50.51	221	380

The average degree of outcross found in this study was 0.77 (ranging from 0 to 1) and its effects, as well as those relative to breed differences on PAP measurements, are presented in Table 3. The estimated regression coefficient for PAP on heterosis was −1.834 ± 1.676 mmHg/percent of outcross (*P* = 0.276), whereas the range of breed effects on PAP was 15.08 mmHg. These results are similar to the regression coefficient of PAP on heterosis of −0.02 ± 1.31 mmHg/percent of outcross and the range of breed effects of 8.75 mmHg reported by Culbertson et al. (2016). In general, the previous imply that heterosis had no effects on PAP measurements and a possible explanation for these results arises from the fact that heterosis effects tend to be inversely proportional to the heritability of a trait (Ritchie et al., 1999). Explicitly, for lowly heritable traits, heterosis benefits are high, whereas for moderately to highly heritable traits (such as PAP), heterosis benefits are expected to be low (Crawford et al., 2016; Kirkpatrick, 2017). The reason for the previous is that lowly heritable traits have a small additive component and, in that scenario, crossbreeding takes advantage of dominance and epistatic effects (Spangler, 2007).

**Table 3.** Estimated values (± SE) and significance level of the linear covariates included in the general linear model

Effect	Estimate	*P*-value
Simmental, %	−2.550 ± 13.210	0.032
Angus, %	4.861 ± 12.440	0.011
Hereford, %	5.346 ± 12.360	0.012
Red Angus, %	12.530 ± 12.050	0.303
Age, d	0.007 ± 0.005	0.228
Degree of outcross, %	−1.834 ± 1.676	0.276

Furthermore, heterotic effects of crossbred individuals are known to be dependent upon the differences in allele frequencies of the loci contributing to variations in the trait, so that the larger these differences, the greater the heterozygosity and its benefits (Kumar et al., 2018). This is relevant since in the present study, more than half of PAP observations belonged to the breed group formed by Angus and Red Angus individuals, a couple of breeds known to have a recent shared ancestry and notable similarities at the genomic level (Bovine HapMap Consortium, 2009). A similar situation could be happening with the breed group conformed by Angus and Hereford animals, since close similarities between these two breeds based on biological values related to growth, ability, and survivability have been reported (Roughsedge et al., 2001). Additionally, despite of the genomic diversity among beef cattle breeds, it has been reported the existence of pleiotropic quantitative trait loci (QTL) that segregate in several phylogenetically distinct breeds (Saatchi et al., 2014). These pleiotropic QTL have been found to be mainly associated with traits for which similar selection pressure has been made within beef cattle breeds (e.g., growth, fertility, and carcass traits). This exemplifies the existence of genomic commonalities across breeds leading to similar performances for economically relevant traits. However, the same could be occurring for the overall cattle susceptibility to the development of HAD, as explained by Holt and Callan (2007).

Finally, when analyzing the estimated regression coefficients for breed percentages, the only reduction in PAP scores corresponding to increments of a breed percentage was for Simmental. For the remaining breeds, the estimated regression lines were positive and similar in magnitude, with the exception of Red Angus for which the greatest increments in PAP scores per breed percentage were observed. These results differ from those presented by Crawford et al. (2017), who reported that among 10 different beef cattle breeds, Simmental bulls were found to have the highest adjusted PAP of the breed groups involved in the study. However, the authors recognized that such result was likely to be associated with the uniqueness of the animals represented in their data, since according to the breed origin at the Simme Valley in Switzerland, Simmentals are thought to tolerate high elevations (American Simmental Association, 2020). In conclusion, no effects of heterosis on PAP were observed in this study, even when the slope of the estimated regression line had a negative tendency. Therefore, we reject our hypothesis that increased levels of heterozygosity would be related to lower PAP scores.
